# All-optical observation and reconstruction of spin wave dispersion

**DOI:** 10.1038/ncomms15859

**Published:** 2017-06-12

**Authors:** Yusuke Hashimoto, Shunsuke Daimon, Ryo Iguchi, Yasuyuki Oikawa, Ka Shen, Koji Sato, Davide Bossini, Yutaka Tabuchi, Takuya Satoh, Burkard Hillebrands, Gerrit E. W. Bauer, Tom H. Johansen, Andrei Kirilyuk, Theo Rasing, Eiji Saitoh

**Affiliations:** 1 WPI Advanced Institute for Materials Research, Tohoku University, Sendai 980-8577, Japan; 2 Institute for Materials Research, Tohoku University, Sendai 980-8577, Japan; 3 Kavli Institute of NanoScience, Delft University of Technology, Lorentzweg 1, Delft 2628 CJ, The Netherlands; 4 Institute for Photon Science and Technology, Graduate School of Science, The University of Tokyo, Tokyo 113-0033, Japan; 5 Research Center for Advanced Science and Technology, The University of Tokyo, Tokyo 153-8904, Japan; 6 Department of Physics, Kyushu University, Fukuoka 819-0395, Japan; 7 Fachbereich Physik and Landesforschungszentrum OPTIMAS, Technische Universität Kaiserslautern, Kaiserslautern 67663, Germany; 8 Department of Physics, University of Oslo, Oslo 0316, Norway; 9 Institute for Superconducting and Electronic Materials, University of Wollongong, Northfields Avenue, Wollongong, New South Wales 2522, Australia; 10 Radboud University Nijmegen, Institute for Molecules and Materials, Nijmegen 6525 AJ, The Netherlands; 11 Advanced Science Research Center, Japan Atomic Energy Agency, Tokai 319-1195, Japan

## Abstract

To know the properties of a particle or a wave, one should measure how its energy changes with its momentum. The relation between them is called the dispersion relation, which encodes essential information of the kinetics. In a magnet, the wave motion of atomic spins serves as an elementary excitation, called a spin wave, and behaves like a fictitious particle. Although the dispersion relation of spin waves governs many of the magnetic properties, observation of their entire dispersion is one of the challenges today. Spin waves whose dispersion is dominated by magnetostatic interaction are called pure-magnetostatic waves, which are still missing despite of their practical importance. Here, we report observation of the band dispersion relation of pure-magnetostatic waves by developing a table-top all-optical spectroscopy named spin-wave tomography. The result unmasks characteristics of pure-magnetostatic waves. We also demonstrate time-resolved measurements, which reveal coherent energy transfer between spin waves and lattice vibrations.

## Abstract (web summary)

Observation of the entire dispersion relation for spin waves remains a challenge which prevents the full understanding of many intriguing magnetic properties. Here, the authors develop a table-top all-optical approach to map out the dispersion curve of pure-magnetostatic waves in magnetic films.

Spin waves (SWs) can be classified into the following three classes: exchange, dipole-exchange and pure-magnetostatic spin waves^1^,^2^,^3^,^4^. Exchange SWs refer to a type of spin waves in which the dispersion relations are dominated by exchange interaction; neutron scattering has been used to measure their dispersion relations^5^. Dipole-exchange SWs refer to a type of spin waves affected both by exchange interaction and magnetostatic dipole interaction; Brillouin light scattering holds an unchallenged position in their observation^6^,^7^,^8^. The third type is pure-magnetostatic SWs whose dispersion is explained without exchange interaction^1^,^2^,^3^,^4^. Spin waves in the magnetostatic regime are characterized by complicated and anisotropic dispersion relations; for instance, their slope may even become negative for the so-called backward volume magnetostatic waves. Although they are currently employed in spintronics and micromagnetics^9^,^10^,^11^,^12^,^13^, a dispersion spectroscopy of pure-magnetostatic waves is still missing; to observe the dispersion requires a new concept in spin-wave spectroscopy.

The most traditional principle of spectroscopy is diffraction caused by periodic structures, such as a grating or a crystal lattice^14^. Many methods of neutron and optical scattering spectroscopy, including Brillouin light scattering, are based on this mechanism^5^,^6^,^7^,^8^,^10^,^13^.

Another powerful principle of spectroscopy is the Fourier transform (FT) method, which has recently become more attractive because of advances in optical- and data-processing technologies as demonstrated by phonon-polariton spectroscopy^15^,^16^,^17^,^18^. In the FT method, all possible waves are excited at once, and power spectra are obtained by calculating the FT of the excited waveform. Such methods have been introduced in some fields including FT infrared spectroscopy (FTIR)^17^ and NMR spectroscopy^15^, and rapidly turned into versatile tools spread all over the world.

In the following, we present spin-wave FT spectroscopy which can reconstruct the dispersion curves of spin waves: we name it spin-wave tomography (SWaT). An ultrashort light pulse is focused on a very small surface area of a magnet to excite spin waves^11^,^19^,^20^,^21^,^22^,^23^,^24^,^25^. When the pulse duration and the excitation area are infinitesimally small, the pulse consists of all temporal and spatial wave components according to the Fourier theorem. Then, spin waves of all wave vectors can be created simultaneously and propagate radially from the excitation point. The created spin waves are detected using a magneto-optical imaging technique^26^ (see Methods section for details). By Fourier transforming the observed propagating waveform with respect to time and spatial coordinates, power spectra of the spin-wave propagation are obtained as a function of the frequency *f* and the wavenumber vector **k**. The spin-wave dispersion relations are then read from the spectra (Supplementary Note 1). The lateral sample size should be larger than the spin-wave decay length to avoid extrinsic standing-wave formation caused by reflection of the spin waves at the sample edges. This method gives access to the spin-wave dispersion with wavelengths typically larger than a micrometre. This is the principle of the SWaT developed here. To obtain the spectra, clear Faraday rotation signals with high spatial resolution are indispensable. Thanks to a recently developed technique to acquire absolute Faraday rotation angles as a function of time and position^26^, we report here the dispersion structures of pure-magnetostatic spin waves observed with SWaT.

## Results

### SWaT measurements

**Figure 1:**
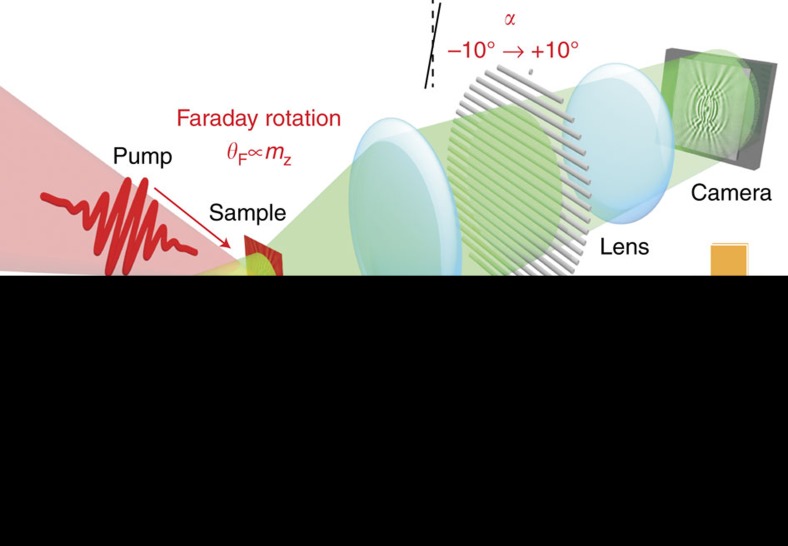
Schematic illustration of spin wave tomography. Spin-wave tomography (SWaT) is based on the observation of the propagation dynamics of spin waves with the pump-and-probe magneto-optical imaging method. Various modes of spin waves are excited simultaneously by the illumination of the pump laser pulse in magnetic materials. The propagation dynamics of the spin waves are measured through the Faraday effect on the probe pulse (*θ*_F_), which is proportional to the magnetization along the direction normal to the sample surface (*m*_z_). The images of *θ*_F_ are obtained by analyzing the transmission images (*I*_α_) observed at various angles of the analyzer (*α*)^26^. The spin-wave dispersion relations are reconstructed by Fourier transforming the propagating waveform of the spin waves.

Figure 1 illustrates our experimental setup. A 100 fs-duration intense laser pump pulse was focused to a small round area (7 μm^2^) of a 4.0 μm-thick LuIG (001) film (see Methods section for details). The pump pulse excites spin waves by photo-magnetic effects, predominantly photo-induced demagnetization^11^,^19^,^22^ and magnetoelasticity^24^,^27^,^28^,^29^ (see below). The temporal evolution of the excited magnetization texture was obtained from the polarization rotation angle of the probe beam, which represents the local magnetization component along the light propagation via the optical Faraday effect^26^,^30^. To acquire temporal evolution data, the time delay *t* between the pump and the probe pulses was scanned. By Fourier transforming the temporal and spatial dependence of the Faraday rotation angle, we obtained power spectra of the Faraday rotation: SWaT power spectra, which reflect the **k**-dependent dynamical magnetic susceptibility^31^ (Supplementary Note 1).

**Figure 2:**
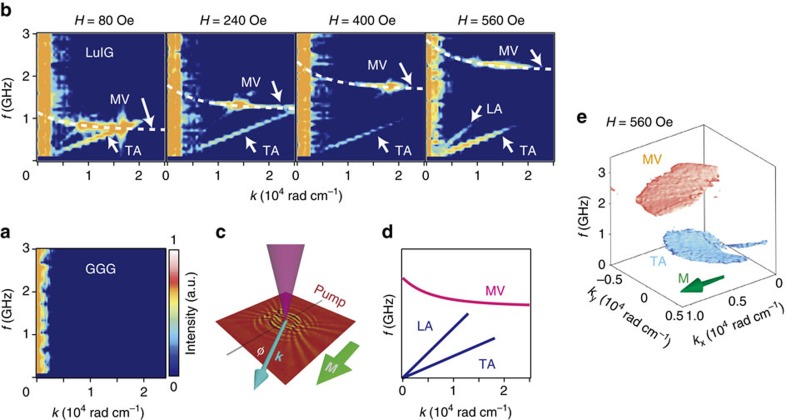
SWaT spectra as a function of external magnetic fields. (**a**) A SWaT spectrum obtained for a paramagnetic GGG substrate without spontaneous magnetization. (**b**) SWaT spectra for the LuIG sample with wavenumber **k** parallel to the magnetization **M**, which was aligned along the [100] axis by an in-plane magnetic field **H**. We observe three different dispersion curves, which are schematically shown in (**d**). TA, LA and MV represent the transverse acoustic phonon, the longitudinal acoustic phonon and the magnetostatic volume mode branches, respectively. The white dashed curves are calculated by a magnetostatic spin wave theory^4^ with the following parameters: saturation magnetization 4π*M*_s_=780 G, cubic anisotropy *K*_c_=2.3 × 10^3^ erg cm^−3^ and uniaxial magnetic anisotropy *K*_u_=−1.2 × 10^4^ erg cm^−3^. (**c**) A schematic of the experimental configuration. *φ* is defined as the angle between **k** of spin waves and the orientation of **M**. (**d**) A schematic of the three branches observed in (**b**). (**e**) A three-dimensional contour plot (at the intensity of 0.7 in the scale shown in (**a**) of the SWaT intensity representing spin waves (red) and phonons (blue). The direction of **M** is indicated by the green arrow.

Figure 2a shows a spectrum for a non-magnetic Gd_3_Ga_5_O_12_ (GGG) crystal that does not carry spin waves obtained by the above SWaT method as a function of the wavenumber *k* along the [100] direction and the frequency *f*. We confirm that no signals appear in the spectrum for this non-magnetic medium, and then move on to measurements on magnetic materials.

In Fig. 2b, we show a SWaT spectrum obtained for a ferrimagnetic LuIG (001) film with a magnetic field (**H**) applied in the [100] direction. Here, **k** is set along the [100] direction (*φ*=0°, where *φ* is defined in Fig. 2c). In Fig. 2b, importantly, clear signals (orange colour) appear around 0.5 GHz-2.0 GHz that are labelled as magnetostatic volume wave (MV) (Fig. 2d,e). The signals shift towards higher frequencies almost proportional to the external magnetic field *H*. This proves that the signals are caused by magnetic excitations.

Notable is that the spectra in Fig. 2b disclose a dispersion curve with negative slope; the signal frequency *f* decreases with increasing *k*, which is a distinct feature of backward volume magnetostatic modes that are expected to be prominent near *k*=0. The experimental signal (orange colour in Fig. 2b) is well reproduced by an analytical expression for backward volume magnetostatic modes^4^ (dashed white curve in Fig. 2b). This is evidence for the observation of pure-magnetostatic spin-wave dispersion curves (Supplementary Movie 1). Higher-order standing spin waves are less visible in the transmission Faraday experiments due to their oscillations along the thickness direction^4^.

In all the spectra measured for LuIG, we observed low-frequency signals, labelled as transverse acoustic (TA) phonon and longitudinal acoustic (LA) phonon in Fig. 2b, each of which exhibits a linear *k* dependence (*f* ∝ *k*). The TA (LA) signal is assigned to transverse (longitudinal) acoustic phonon dispersion, since its slope, *v*=3.0 km s^−1^ (6.2 km s^−1^), is consistent with the transverse (longitudinal) sound velocity^32^. This means that low-frequency lattice vibrations are coupled to magnetization oscillations, and the present method is sufficiently sensitive to pick them up.

### Angular dependence of SWaT

**Figure 3:**
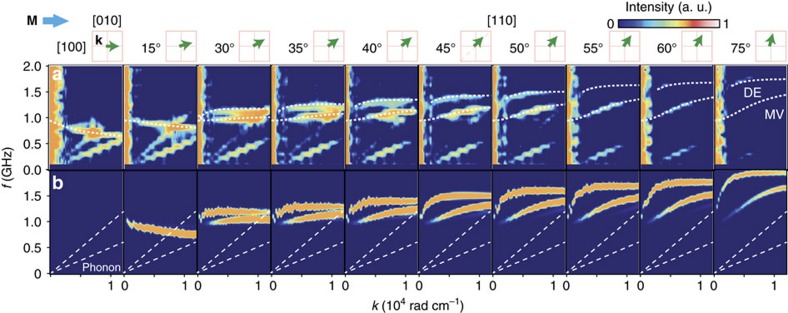
Angular dependence of SWaT spectra. (**a**) Experimentally obtained angular dependence of the SWaT spectra for various **k** directions. The angle (*φ*) between **k** and **M** is shown at the top of each figure. The branches of spin waves labelled as DE and MV represent the magnetostatic Damon–Eshbach and magnetostatic volume modes, respectively. The white dashed curves are calculated by magnetostatic spin wave theory^4^. (**b**) Spin-wave dispersion obtained by a micromagnetic simulation (see text) with the same parameters as in (**a**). The white dashed lines represent the phonon dispersion.

In Fig. 3a, we show the **k**-direction dependence of the obtained SWaT spectra for LuIG (Supplementary Movie 1). With changing the direction of **k**, the dispersion drastically changes, again consistent with magnetostatic spin wave theory^4^. The slope of the MV dispersion changes from negative to positive when the angle (*φ*) between **k** and **M** (Fig. 2c) is around 30°. This is caused by the uniaxial magnetocrystalline anisotropy along the surface normal; the experimental results are well reproduced by a theoretical calculation^4^ including this uniaxial anisotropy, shown as the white dashed curves in Fig. 3a. The dispersion signal labelled as DE in Fig. 3a can be assigned to a spin wave called a magnetostatic Damon-Eshbach mode^4^. The dispersion curves of MV and DE show negative and positive slopes, respectively, consistent with microwave absorption data^33^. For comparison, in Fig. 3b, we show numerically calculated power spectra of the pure-magnetostatic spin waves for the **k** directions in Fig. 3a; the temporal evolution of the out-of-plane magnetization distribution generated by a sudden change in the magnetization at the origin was calculated by solving the Landau–Lifshitz–Gilbert equation^34^. The parameters are the same as those used for the fits in Fig. 3a. The measured dispersion curves are well reproduced by the calculations for all **k** directions. This demonstrates again that the observed dispersion signals pertain to pure-magnetostatic spin waves, and that SWaT works properly and is powerful enough to directly map them.

### Time-resolved SWaT

Finally, we present time-resolved spectra that provide information about the spin-wave excitation mechanism and the role of phonons. Time-resolved SWaT spectra were obtained by clipping out data over a time interval, Δ_t_, centred around a time, *t*_c_, using a Gaussian window function and performing the SWaT analysis for the clipped data (Supplementary Note 2). Here, we set Δ_t_ as 2.8 ns corresponding to the frequency resolution of ∼0.4 GHz.

**Figure 4:**
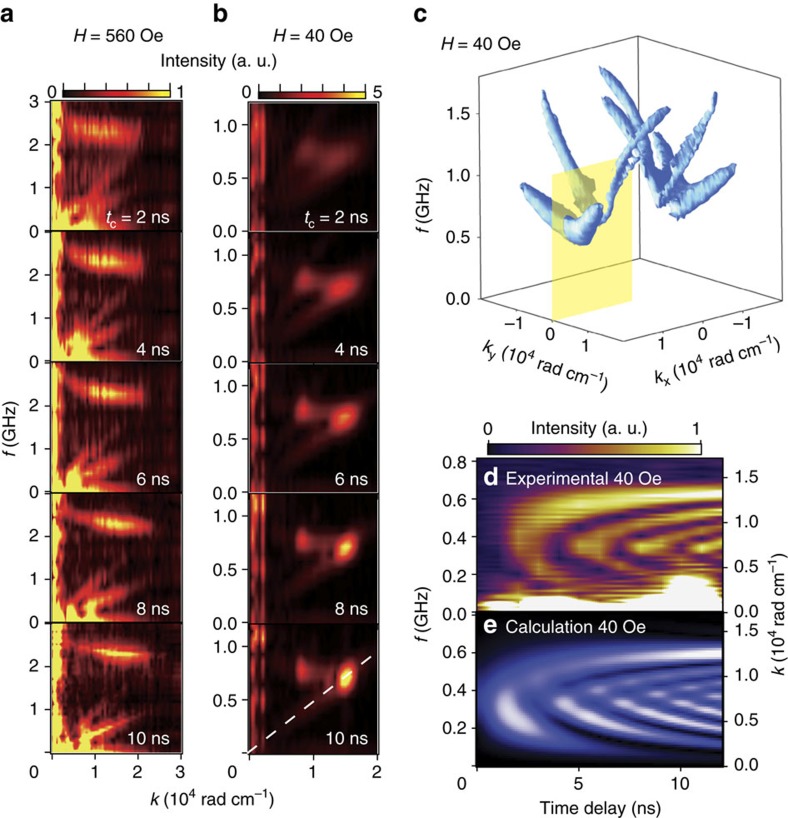
Time-resolved SWaT spectra. (**a**,**b**) Time-resolved SWaT spectra with **k** parallel to **M** under in-plane magnetic fields of 560 Oe (**a**) and 40 Oe (**b**) respectively. The data was obtained by applying a time-window with the width of 2.8 ns centred at the time delay noted in each figure. (**c**) A three-dimensional contour plot (at the intensity of 0.97 in the scale shown in Fig. 2a) of the SWaT intensity obtained under the in-plane magnetic field of 40 Oe. The data shown in (**b**) was extracted along the cross-section shown as the yellow plane. (**d**) Temporal evolution of the time-resolved SWaT spectra obtained along the dashed white line in (**b**). The data was obtained by applying a time-window with the width of 1.0 ns. (**e**) A result of numerical calculations of the time-resolved SWaT spectra shown in (**d**).

We observed significant difference in the time evolution of the spectral intensity under the external magnetic fields of 560 Oe (Fig. 4a) and 40 Oe (Fig. 4b,c). This is attributed to the difference in the dominant spin-wave excitation mechanisms: the photo-induced demagnetization or the magnetoelastic coupling. Figure 4a shows time-resolved SWaT spectra for LuIG under the magnetic field of 560 Oe (see also Supplementary Movie 1). The spin wave branches are already recognizable in the spectra at *t*_c_=2 ns. At this magnetic field, the spin waves appear to be excited mainly by the photo-induced demagnetization process^11^,^19^,^22^; the spin wave spectral intensity is unchanged when the pump pulse polarization is changed among clockwise, anticlockwise and linear, excluding the possibility of the inverse Faraday effect^21^,^29^,^35^,^36^ or photo-induced magnetic anisotropy^23^ as excitation mechanisms. The demagnetizing field is changed by the laser light in the pumped area, and the resulting impulse can contribute to the spin-wave emission^11^,^19^,^22^. At *H*=40 Oe, on the other hand, the spectral intensity at *k*∼0.8 × 10^4^ rad cm^−1^ and *k* ∼ 1.5 × 10^4^ rad cm^−1^ in the spectra gradually increases over time (Fig. 4b). This implies an energy transfer from phonons (LA and TA) to spin waves via the crossing points of their dispersion curves. At these points, the spin waves can be excited directly by phonons via magnetoelastic coupling (Supplementary Note 3), known as magnetoelastic waves, although phonons are less visible in the Faraday rotation. The difference in the dominant excitation mechanisms in Fig. 4a,b is due to the strong magnetic field dependence in the magnetoelastic waves, of which the intensity significantly reduces with increasing the external field (Supplementary Note 4). Consequently, spin waves are dominantly generated by the photo-induced demagnetization at 560 Oe, while the magnetoelastic coupling is the dominant mechanism at 40 Oe.

Figure 4d shows the temporal evolution of the SWaT spectral intensity along the dashed line in Fig. 4b for 10 ns. Interestingly, a streak pattern appears below the dispersion-crossing frequency of ∼0.7 GHz. This streak is attributed to the beat pattern between spin-wave and phonon modes, which interfere near the intersection point while satisfying momentum and energy conservation (Supplementary Note 3). In fact, a numerical calculation shown in Fig. 4e, in which the Landau–Lifshitz–Gilbert equation is solved combined with the magnetoelastic coupling to transverse acoustic phonons, well reproduces the observed streak pattern.

## Discussion

Finally, let us discuss the advantages of SWaT. As we demonstrated, SWaT can be employed as a new technique for the dispersion spectroscopy of pure-magnetostatic waves. The SWaT is a non-contact and non-destructive spectroscopy for magnetic excitation and phonons that can be applied even to minute and thin samples. SWaT measurements can also be made under various environments, such as strong electric fields, strong magnetic fields, varying temperature and high pressure. As a result of this versatility, SWaT might find potential applications in the evaluation of the spin-wave dispersion not only in magnetic materials, but also in, for instance, topological insulators and magnetic films attached on superconductors. The obtained dispersion of spin waves would form the basis for the development of spintronics and magnonics devices. Moreover, time-resolved measurements of SWaT may be utilized for the real-time imaging of interaction between fundamental excitations in solids, like electronic quasiparticles, phonons and spin waves.

## Methods

### Sample

We used a 4.0 μm-thick film of Bi-doped iron garnet Lu_2.3_Bi_0.7_Fe_4.2_-Ga_0.8_O_12_ (LuIG) grown on a Gd_3_Ga_5_O_12_ (001) substrate, which is a well-established material for magneto-optical imaging^37^,^38^. The saturation magnetization of the sample (4π*M*_s_) was measured to be 780 G using a vibrating sample magnetometer. The magneto-optical properties of LuIG films were reported in refs 37, 38. At the wavelength of the probe pulse (630 nm), the sample shows a large Faraday rotation angle of 5.2 degrees and a high transmissivity of 41%. All the measurements were performed at room temperature.

### Ultrafast time-resolved magneto-optical imaging

Propagation dynamics of the optically-excited spin waves were observed using an ultrafast time-resolved magneto-optical imaging system with sub-picosecond time resolution, sub-micrometre spatial resolution and millidegree resolution in the light polarization rotation angle^26^. This system, schematically represented in Fig. 1, is based on an all-optical two-colour pump-probe technique for the time-resolved measurements in combination with a rotation analyser method to obtain magneto-optical images. As a light source, we used a 100 fs amplified laser system with the center wavelength of 800 nm and a repetition frequency of 1 kHz. The output of the laser was divided into two beams: the pump and the probe beams. The beam with the original wavelength (800 nm) was used for the pump. The probe wavelength was tuned with an optical parametric amplifier to 630 nm. The pump pulse was circularly polarized and focused to a small round area (7 μm^2^) on the sample surface. The probe beam illuminated the sample with a focus size of ∼1 mm. The probe fluence (0.2 mJ cm^−2^) was much weaker than the pump fluence (1.2 J cm^−2^). To measure the photo-induced change in the magnetization distribution, we measured light polarization images of the transmitted probe beam using the rotation analyzer method, composed of an analyzer (a Glan-Taylor prism) and a CCD (charge-coupled device) camera. The analyzer angle (*α*) was controlled with a motorized rotation stage. For the measurements, we scan *α* from −10 degrees to 10 degrees, and the CCD camera measures the light intensity images of the transmitted probe beam as a function of *α*, which we denote as *I*(*α*). Furthermore, *I*(*α*) is given by *I*(*α*)=*I*_t_sin^2^ (*θ*_F_*−α*)+*I*_b_, where *I*_t_, *θ*_F_ and *I*_b_ represent the transmitted light intensity, the Faraday rotation angle and the background signal, respectively. By fitting the obtained *I*(*α*) data with this equation, we obtained three images of *I*_t_, *θ*_F_ and *I*_b_ simultaneously. The quantity *θ*_F_ reflect the magnetic properties of the sample. SWaT spectra were obtained by performing spatial and temporal FT of the *θ*_F_ data set. A Hanning window was employed for the FT along the time axis to reduce noise.

### Data availability

The data that support the findings of this study are available from the corresponding author on request.

## Additional information

**How to cite this article:** Hashimoto, Y. *et al*. All-optical observation and reconstruction of spin wave dispersion. *Nat. Commun.* **8,** 15859 doi: 10.1038/ncomms15859 (2017).

**Publisher's note:** Springer Nature remains neutral with regard to jurisdictional claims in published maps and institutional affiliations.

## Supplementary Material

Supplementary Information Supplementary Notes and Supplementary References

Supplementary Video This supplementary movie demonstrates two data. One is the angular dependence of the SWaT spectra obtained at in-plane magnetic field of 40 Oe. Another is the temporal changes of two SWaT spectra obtained at in-plane magnetic elds of 40 Oe and 560 Oe.

Peer Review file
