# Supplementary material for: All-optical observation and reconstruction of spin wave dispersion
Source: Nat Commun. 2017 Jun 12;8:15859. doi: 10.1038/ncomms15859 (PMC5477491; doi:10.1038/ncomms15859)
Supplement: Supplementary Information — Supplementary Notes and Supplementary References [file ncomms15859-s1.pdf]

SUPPLEMENTARY NOTE 1: DETERMINATION OF DISPERSION RELATION OF SPIN WAVES

We show how the spin-wave tomography (SWaT) captures the dispersion relation of spin waves. It is known that the dispersion relation of spin waves is determined by the dynamical susceptibility [1]. In SWaT, we determine the dynamical susceptibility of the magnet by experimentally observing the propagation dynamics of spin waves, which are generated by the illumination of a pulsed and focused light. More detailed discussions are given below.

In SWaT, spin waves are triggered by the pump pulse illumination, inducing various effective fields inherent to the system: a dipolar field and an anisotropy field. Given an appropriate expression of free energy $F_{\rm ext}[\mathbf{M}]$, which includes the effects induced by the pump pulse illumination, the effective field is found by

$$\mathbf{H}'(\mathbf{r}) = -\frac{\delta F_{\rm ext}[\mathbf{M}]}{\delta \mathbf{M}(\mathbf{r})} \,. \tag{1}$$

Treating $\mathbf{H}'$ as a perturbation, the leading order response of $\mathbf{M}(\mathbf{r}, t)$ to $\mathbf{H}'$ is given by

$$M_i(\mathbf{r}, t) = \int d\mathbf{r}' \int_{-\infty}^{\infty} dt' \chi^R_{ij}(\mathbf{r} - \mathbf{r}', t - t') H'_j(\mathbf{r}', t') \,, \tag{2}$$

where $\chi^R_{ij}(\mathbf{r}, t)$ is the retarded nonlocal susceptibility describing a response to H'_j with $i, j = x, y, z$. Here, we extract the information of $\chi^R_{ij}(\mathbf{r}, t)$ by experimentally observing the propagation dynamics of spin waves via the magneto-optical signal $\Theta_{\mathrm{MO}}(\mathbf{r}, t)$, which is the Faraday rotation angle in our experiment and related to the out-of-plane component of the magnetization $M_z(\mathbf{r}, t)$ [2].

The Fourier transform of Supplementary Eq. (2) from the space-time domain to the wavenumber-frequency domain $(\mathbf{k}, \omega)$ gives

$$M_i(\mathbf{k}, \omega) = \chi^R_{ij}(\mathbf{k}, \omega) H'_j(\mathbf{k}, \omega)\,, \tag{3}$$

where $M_i(\mathbf{k}, \omega) = \mathscr{F}\{M_i(\mathbf{r}, t)\}$, $\chi^R_{ij}(\mathbf{k}, \omega) = \mathscr{F}\{\chi^R_{ij}(\mathbf{r}, t)\}$, and $H'_i(\mathbf{k}, \omega) = \mathscr{F}\{H'_i(\mathbf{r}, t)\}$ with $\mathscr{F}$ denoting the Fourier transform. It is known that the imaginary part of the susceptibility, $\mathrm{Im}\chi_{ij}(\mathbf{k}, \omega)$, is proportional to the spin spectral function, which encodes the information of the dispersion relation of spin waves [1]. Therefore, observing the structure of $M_z(\mathbf{k}, \omega)$ by SWaT, we can extract the dispersion relation of spin waves.

In our experiment, $H'_j(\mathbf{k}, \omega)$ is induced by the pump pulse illumination of the magnet via various processes, including inverse Faraday effect [3], photo-induced demagnetization [4, 5], photo-induced magnetic anisotropy [6] and magnetoelasticity [7]. Below we show three cases with different forms of $H'_j(\mathbf{k}, \omega)$.

First, we consider an impulsive effective field induced by the pump pulse illumination. Approximating the spatial and temporal distributions of the pump pulse by delta functions, the effective field is expressed by $H_j'^{\text{delta}}(\mathbf{r},t) = F_j\delta(\mathbf{r})\delta(t)$, where F_j is proportional to the photo-induced effective field. Since its Fourier transform is given by $\mathscr{F}\{H_j'^{\text{delta}}(\mathbf{r},t)\} = F_j$, Supplementary Eq. (3) leads to $M_z(\mathbf{k},\omega) = \chi_{zj}^R(\mathbf{k},\omega)F_j$. Therefore, the SWaT spectrum captures $\chi_{zj}^R(\mathbf{k},\omega)$, weighted by F_j. The impulsive stimulated Raman scattering is modeled by this case [3].

Next, we consider the effective field which is generated by the pump pulse illumination and then stays constant. This field is expressed by $H_j'^{\text{step}}(\mathbf{r},t) = F_j\delta(\mathbf{r})\Theta(t)$, where $\Theta(t)$ is a step function. In this case, the Fourier transform of $M_z(\mathbf{r},t)$ is given by $M_z(\mathbf{k},\omega) = \left(\pi\delta(\omega) + i\frac{1}{\omega}\right)\chi_{zj}^R(\mathbf{k},\omega)F_j$. Thus, the SWaT spectrum for $\omega \neq 0$ captures $\chi_{zj}^R(\mathbf{k},\omega)$, weighted by iF_j/ω. The photo-induced demagnetization [4, 5] and the photo-induced magnetic anisotropy [6] can be modeled by this effective field.

Finally, we consider the effective field of the form $\mathbf{H}'(\mathbf{r},t) = \Theta(t)\tilde{\mathbf{H}}'(\mathbf{r},t)$, where $\tilde{\mathbf{H}}'(\mathbf{r},t)$ describes the contributions induced by the coupling between spin waves and other excitations, such as phonons interacting with spin waves through the magnetoelastic coupling [7]. In this case, $\mathbf{H}'(\mathbf{k},\omega) = \mathscr{F}\{\mathbf{H}'(\mathbf{r},t)\}$ is expressed in terms of the propagators of these excitations and also reflects their dispersion relations.

Therefore, examining the peak structure of $M_z(\mathbf{k}, \omega)$ in Supplementary Eq. (3) reveals the information of the dispersion relations of spin waves and other relevant excitations.

Supplementary note 2: Time-Frequency Analysis

We extract the temporal evolution of the SWaT spectra by using the Gapor transform (GT), which is a Fourier transform in a finite time window. The GT of the function $h(t)$ is defined as

$$\mathcal{G}(\omega, t_c) = \int_{-\infty}^{\infty} e^{-i\omega t} h(t) g(t - t_c) dt \,, \tag{4}$$

where $g(t)$ is a time window function and $h(t)$ represents the measured data. t_c is the central time of the time window. In the present study, a Gaussian window,

$$g(t) = \frac{1}{\Delta_t \sqrt{\pi}} e^{-(t/\Delta_t)^2} \,, \tag{5}$$

was used. The temporal evolution of the SWaT spectrum is obtained by calculating $\mathcal{G}(\omega, t_c)$ with different values of t_c in Supplementary Eq. (4). The results are shown in Figs. 4(a) and 4(b) for external fields of 560 Oe and 40 Oe, respectively, and $\Delta_t = 2.8$ ns. The data shown in Fig. 4(d) was obtained by using GT with $\Delta_t = 1$ ns.

Supplementary note 3: Dynamics of spin waves by optically-excited phonons via magnetoelastic coupling

In this section, we explain the calculations to obtain Fig. 4(e), which corresponds to the time evolution of the SWaT spectra of spin waves excited by the heat induced phonons via the magnetoelastic coupling. We assume that the pump pulse locally heats the magnetic layer. The time evolution of the temperature field under the influence of the pump pulse can be modeled as

$$T(\mathbf{r},t) = T_0 + \Delta T e^{-r^2/W^2}\Theta(t), \tag{6}$$

where W is the effective heating (laser spot) area. T_0 is the initial temperature before the pump pulse irradiation, and $\Theta(t)$ is the step function. The heat from the pump pulse does not leave the pumped area by heat diffusion, which we estimate to spread the heat spot by only 20 nm in the time scale of the experiment, because the thermal conduction is small enough to prevent heat diffusion. However, the spot loses energy by the emission of ballistic phonons and spin waves.

After the heating process, the lattice starts to release shear and pressure stress due to temperature gradients, and acoustic phonons are excited as a consequence. In principle, the longitudinal and transverse displacements are mixed near the surface of the sample, leading to surface acoustic waves. Here, we can neglect such mixing

and focus on the dynamics of the transverse displacement (denoted by R_z) due to the heat induced shear stress, which is found to be the dominant contribution in our experiment. The equation of motion for the out-of-plane transverse displacement reads

$$\partial_t^2 R_z(\mathbf{r},t) - c_t^2 \nabla^2 R_z(\mathbf{r},t) = \eta \nabla^2 T(\mathbf{r},t)\,, \tag{7}$$

where c_t stands for the transverse sound velocity. The right hand side of Supplementary Eq. (7) is proportional to the heat induced shear stress exerted on the lattice, and η is a phenomenological parameter proportional to the thermal expansion coefficient. By solving Supplementary Eq. (7) in the Fourier space, the temporal evolution of this shear wave is

$$R_z(\mathbf{k},t) \;\; = \;\; \int \frac{d\omega}{2\pi} e^{i\omega t} \frac{\eta k^2}{(\omega + c_t k)(\omega - c_t k)\omega} e^{-W^2 k^2/4}. \tag{8}$$

Note that a given phonon mode propagates in the xy plane, namely $\mathbf{k}$ has only x and y components, and the depth dependence of R_z is disregarded for simplicity.

Including the magnetoelastic coupling due to R_z, external field $\hat{\mathbf{e}}_x H_0$, uniaxial anisotropy field $-(2K_u m_z/M_s)\hat{\mathbf{e}}_z$, cubic anisotropy field $(2K_c/M_s)(m_y \hat{\mathbf{e}}_y + m_z \hat{\mathbf{e}}_z)$, and dipolar field $\mathbf{h}$, where we let $\mathbf{M} = M_s \mathbf{m}$ with M_s being the saturation magnetization, the linearized Landau-Lifshitz equation around the applied field direction

(x-direction) reads [8]

$$\partial_t \begin{pmatrix} m_y \\ m_z \end{pmatrix} = \begin{pmatrix} 0 & -\omega'_H \\ \omega_H & 0 \end{pmatrix} \begin{pmatrix} m_y \\ m_z \end{pmatrix} + \gamma \begin{pmatrix} 0 & 1 \\ -1 & 0 \end{pmatrix} \begin{pmatrix} h_y \\ h_z \end{pmatrix} + \tau_{\mathrm{ME}} \,, \tag{9}$$

Here, we define $\omega_H = \gamma(H_0 + \frac{2K_c}{M_s})$, and $\omega_{H'} = \gamma(H_0 + \frac{2K_c}{M_s} - \frac{2K_u}{M_s})$, where $\gamma > 0$ is the gyromagnetic ratio. In Supplementary Eq. (9), τ_{ME} is the time dependent effective torque arising from the magnetoelastic coupling and given by

$$\tau_{\mathrm{ME}}(\mathbf{r}, t) = \zeta \partial_x R_z(\mathbf{r}, t) \begin{pmatrix} 1 \\ 0 \end{pmatrix} \,, \tag{10}$$

where ζ is the magnetoelastic coupling constant. Its Fourier components in the wave number-time domain is thus given by

$$\tau_{\mathrm{ME}}(\mathbf{k}, t) \;\; = \;\; i\zeta k \cos\theta_{\mathbf{k}} R_z(\mathbf{k}, t) \begin{pmatrix} 1 \\ 0 \end{pmatrix} \tag{11}$$

for a given in-plane wave vector $\mathbf{k} = (k_x, k_y) = k(\cos\theta_{\mathbf{k}}, \sin\theta_{\mathbf{k}})$ at time t, where $\theta_{\mathbf{k}}$ is the angle between $\mathbf{k}$ and and the direction of the magnetic field (x-direction). By solving Supplementary Eq. (9) without the torque term, the following transcendental

equation is obtained [9]:

$$(1+\kappa)\left[1+\tilde{\kappa}\sin^2\theta_{\mathbf{k}}+2\sqrt{-\frac{1+\tilde{\kappa}\sin^2\theta_{\mathbf{k}}}{1+\kappa}}\cot(k_z^i d)\right]+1-\nu^2\sin^2\theta_{\mathbf{k}}=0\,, \tag{12}$$

where $\kappa = \frac{\omega_H\omega_M}{\omega_H\omega_{H'}-\omega_{n\mathbf{k}}^2}$, $\tilde{\kappa} = \frac{\omega_{H'}\omega_M}{\omega_H\omega_{H'}-\omega_{n\mathbf{k}}^2}$, $\nu = \frac{\omega_{j\mathbf{k}}\omega_M}{\omega_H\omega_{H'}-\omega_{n\mathbf{k}}^2}$, and $k_z^i = k\sqrt{-\frac{1+\tilde{\kappa}\sin^2\theta_{\mathbf{k}}}{1+\kappa}}$ with $\omega_M = \gamma M_s$. Here, d is the thickness of the magnetic film. We adopt the electromagnetic boundary conditions for magnetic films with finite thickness from Ref. [9]. From Supplementary Eq. (12), we can obtain the dispersion relation of the spin wave $\omega_{n\mathbf{k}}$ with the mode index $n = 1, 2, 3, \cdots$, which is consistent with our experimental observations and the result of the numerical calculations (Figs. 4d and 4e). To proceed, it is useful to express the torque term in Supplementary Eq. (11) in terms of the eigenfunctions of spin waves as

$$\tau_{\mathrm{ME}}(\mathbf{k},t) = i\zeta k\cos\theta_{\mathbf{k}}R_z(\mathbf{k},t)\sum_n a_{n\mathbf{k}}\begin{pmatrix}(m_{n\mathbf{k}})_y\\ \\ (m_{n\mathbf{k}})_z\end{pmatrix}\,, \tag{13}$$

and we set $\sum_n a_{n\mathbf{k}}(m_{n\mathbf{k}})_y \equiv 1$ and $\sum_n a_{n\mathbf{k}}(m_{n\mathbf{k}})_z \equiv 0$ accordingly to Supplementary Eq. (11). From these conditions, we numerically calculated the wave function $(m_{n\mathbf{k}})_y(z)$ and $(m_{n\mathbf{k}})_z(z)$ of n-th mode from magnetostatic theory and the factor $a_{n\mathbf{k}}$.

All excited spin waves generated before the measurement time t contribute to the Faraday angle, which is proportional to m_z,

$$\Theta_{\mathrm{MO}}(\mathbf{k},t) \propto i\zeta k\cos\theta_{\mathbf{k}}\sum_n\int_0^t dt' e^{-i\omega_{n\mathbf{k}}(t-t')}a_{n\mathbf{k}}\left(m_{n\mathbf{k}}\right)_z R_z(\mathbf{k},t')\,, \tag{14}$$

where $e^{-i\omega_{n\mathbf{k}}(t-t')}$ includes the phase due to the magnetization precession. By substituting Supplementary Eq. (8), we obtain

$$\begin{aligned}\Theta_{\mathrm{MO}}(\mathbf{k},t\geq 0) &\propto i\zeta\eta k e^{-W^2k^2/4}\cos\theta_{\mathbf{k}}\sum_n a_{n\mathbf{k}}\left(m_{n\mathbf{k}}\right)_z \\ &\quad\times\left[2\frac{e^{-i\omega_{n\mathbf{k}}t}-1}{\omega_{n\mathbf{k}}}-\frac{e^{-i\omega_{n\mathbf{k}}t}-e^{-ic_tkt}}{\omega_{n\mathbf{k}}-c_tk}-\frac{e^{-i\omega_{n\mathbf{k}}t}-e^{ic_tkt}}{\omega_{n\mathbf{k}}+c_tk}\right].\end{aligned} \tag{15}$$

As expected, Θ_{MO} vanishes at $t=0$. The square bracket in Supplementary Eq. (15) reveals three-types of responses, namely a static one ($\omega=0$), one at magnon frequencies ($\omega_{n\mathbf{k}}$), and a third one at phonon frequencies ($\pm c_t k$). The last ones are visible because the shear stress acts as an AC driving force for the magnetization dynamics. Once the frequency of the driving force matches the intrinsic frequency of the magnetic system, spin waves are resonantly excited, which is explained by the second and third terms in Supplementary Eq. (15). The GT of Supplementary

Eq. (15), with the central time t_c and the resolution parameter Δ_t, becomes

$$\Theta_{\mathrm{MO}}(\mathbf{k},\omega,t_c) \propto i\zeta\eta k e^{-W^2k^2/4}\cos\theta_{\mathbf{k}}\sum_n a_{n\mathbf{k}}\left(m_{n\mathbf{k}}\right)_z e^{i\omega t_c} \times\left[-\frac{2c_t^2k^2e^{-i\omega_{n\mathbf{k}}t_c}e^{-\Delta_t^2(\omega_{n\mathbf{k}}-\omega)^2/4}}{\omega_{n\mathbf{k}}\left(\omega_{n\mathbf{k}}-c_tk\right)\left(\omega_{n\mathbf{k}}+c_tk\right)}-\frac{2e^{-\Delta_t^2\omega^2/4}}{\omega_{n\mathbf{k}}} +\frac{e^{-ic_tkt_c}e^{-\Delta_t^2(\omega-c_tk)^2/4}}{\omega_{n\mathbf{k}}-c_tk}+\frac{e^{ic_tkt_c}e^{-\Delta_t^2(\omega+c_tk)^2/4}}{\omega_{n\mathbf{k}}+c_tk}\right]. \quad (16)$$

Figure 4(d) is calculated along the transverse phonon branch by setting $\omega = c_tk$ with $\Delta_t = 1\,\mathrm{ns}$.

Supplementary note 4: Magnetic field dependence of magnetoelastic waves

In the SWaT spectrum obtained at 40 Oe (Fig. 4b), we observed strong spectral intensity at the crossing point of the dispersion curves of spin waves and phonons. This is due to spin waves resonantly excited by photo-induced phonons via the magnetoelastic coupling (MEC), known as magnetoelastic waves. Under higher external field, we find that the spectral intensity at the crossing point decreases, as seen in the data of 560 Oe (Fig. 4a). This trend is caused by the combination of two effects, which are the k-dependence of the effective field induced by MEC and the shift of the crossing point by the external field as discussed in the following.

The photo-induced effective field via MEC is proportional to $k\exp(-k^2r_0^2)$, because MEC is proportional to k [10] and the intensity of the pump pulse in k-space is proportional to $\exp(-k^2r_0^2)$, where r_0 is the radius of the pump focus on the sample surface. This function of $k\exp(-k^2r_0^2)$ has a maximum near $k \sim 0.5 \times 10^4$ [rad/cm] when $r_0 = 1.5$ μm and monotonically decreases with increasing k for sufficiently large k. Note that the crossing points for both 40 Oe and 560 Oe applied fields occur in such region of k. Therefore, the effective field induced by MEC decreases with increasing k. With increasing the strength of the external field, the crossing point shifts toward higher k values since the dispersion branches of spin waves are shifted toward higher frequency under stronger fields. Therefore, due to the combination of these two effects, the effective field induced by MEC decreases as the external field is increased.

Supplementary references

[1] White, R. M. *Quantum Theory of Magnetism: Magnetic Properties of Materials.* (Springer, New York, 2007).

[2] Kotov, V. A. & Zvezdin, A. K.. *Modern Magnetooptics and Magnetooptical Materials.* (Institute of Physics Publishing, London, 1997).

[3] Kalashnikova, A. M., Kimel, A. V., Pisarev, R. V., Gridnev, V., Usachev, P., Kirilyuk, A., & Rasing, Th. Impulsive excitation of coherent magnons and phonons by subpicosecond laser pulses in the weak ferromagnet FeBO3. *Physical Review B* **78**, 104301 (2008).

[4] van Kampen, M., *et al*. All-Optical Probe of Coherent Spin Waves. *Physical Review Letters* **88**, 227201 (2002).

[5] Lenk, B. Ulrichs, H. Garbs, F. & Münzenberg, M. The building blocks of magnonics. *Physics Reports* **507**, 107-136 (2011).

[6] Yoshimine, I. *et al*. Phase-controllable spin wave generation in iron garnet by linearly polarized light pulses. *Journal of Applied Physics* **116**, 043907 (2014).

[7] Ogawa, N. *et al*. Photodrive of magnetic bubbles via magnetoelastic waves. *Proceedings of the National Academy of Sciences* **112**, 8977 (2015).

[8] Shen, K & Bauer, G. E. W. Laser-Induced Spatiotemporal Dynamics of Magnetic Films. *Physical Review Letters* **115**, 197201 (2015).

[9] Hurben, M. J. & Patton, C. E. Theory of magnetostatic waves for in-plane magnetized anisotropic films. *Journal of Magnetism and Magnetic Materials* **163**, 39-69 (1996).

[10] Kittel. C, Interaction of spin waves and ultrasonic waves in ferromagnetic crystals. *Physical Review* **110**, 836-841 (1958).
