# Supplementary material for: All-optical observation and reconstruction of spin wave dispersion
Source: Nat Commun. 2017 Jun 12;8:15859. doi: 10.1038/ncomms15859 (PMC5477491; doi:10.1038/ncomms15859)
Supplement: Peer Review file [file ncomms15859-s3.pdf]

Editorial Note:

This manuscript has been previously reviewed at another journal that is not operating a transparent peer review scheme. This document only contains reviewer comments and rebuttal letters for versions considered at Nature Communications.
